# Supplementary figures and images for: Genome-Wide Identification of DUF668 Gene Family and Expression Analysis under Drought and Salt Stresses in Sweet Potato [Ipomoea batatas (L.) Lam]
Source: Genes (Basel). 2023 Jan 14;14(1):217. doi: 10.3390/genes14010217 (PMC9858669; doi:10.3390/genes14010217)

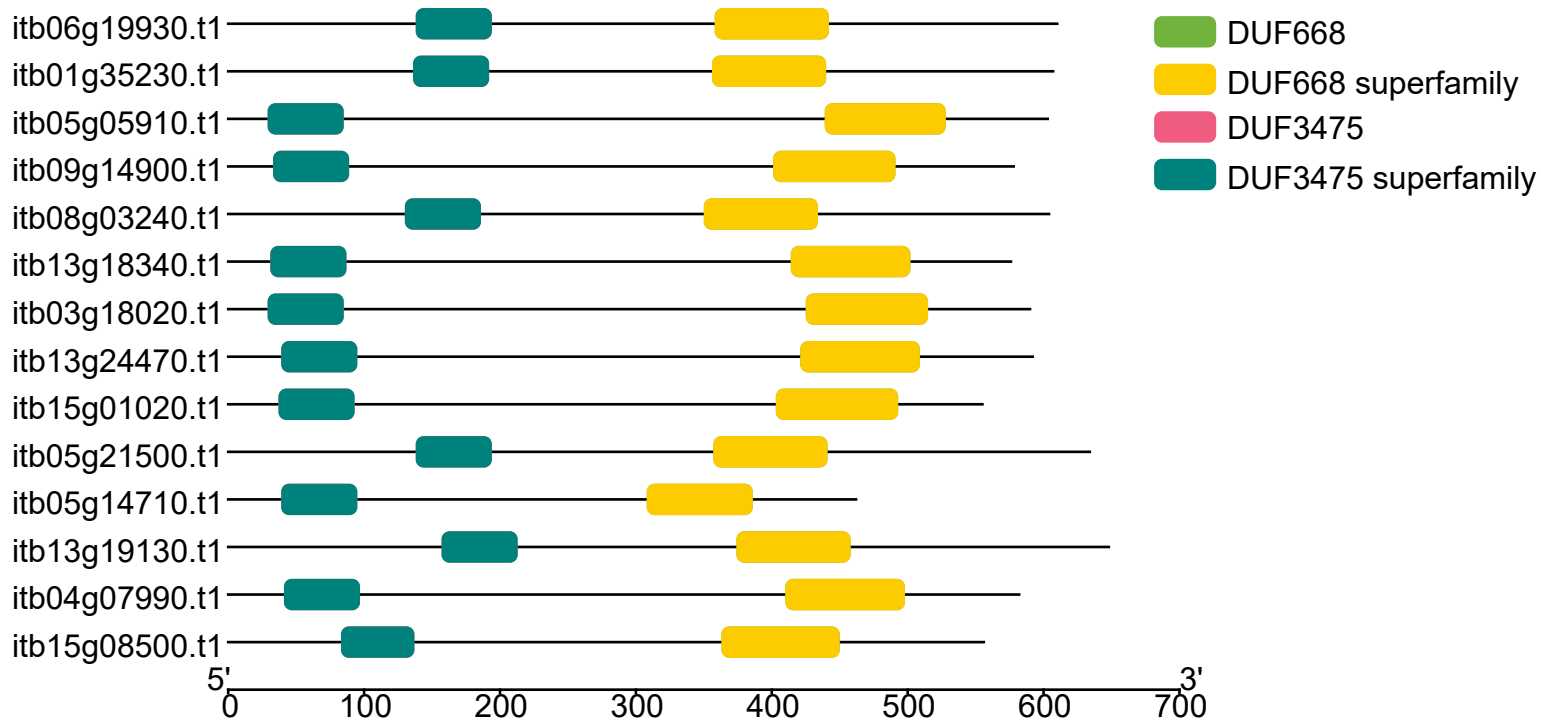

Figure S1: Distribution of the DUF668 domain in the DUF668 proteins of *Ipomoea batatas*

Supplement: Supplementary file 1 [file genes-14-00217-s001.zip › Figure S1.pdf]

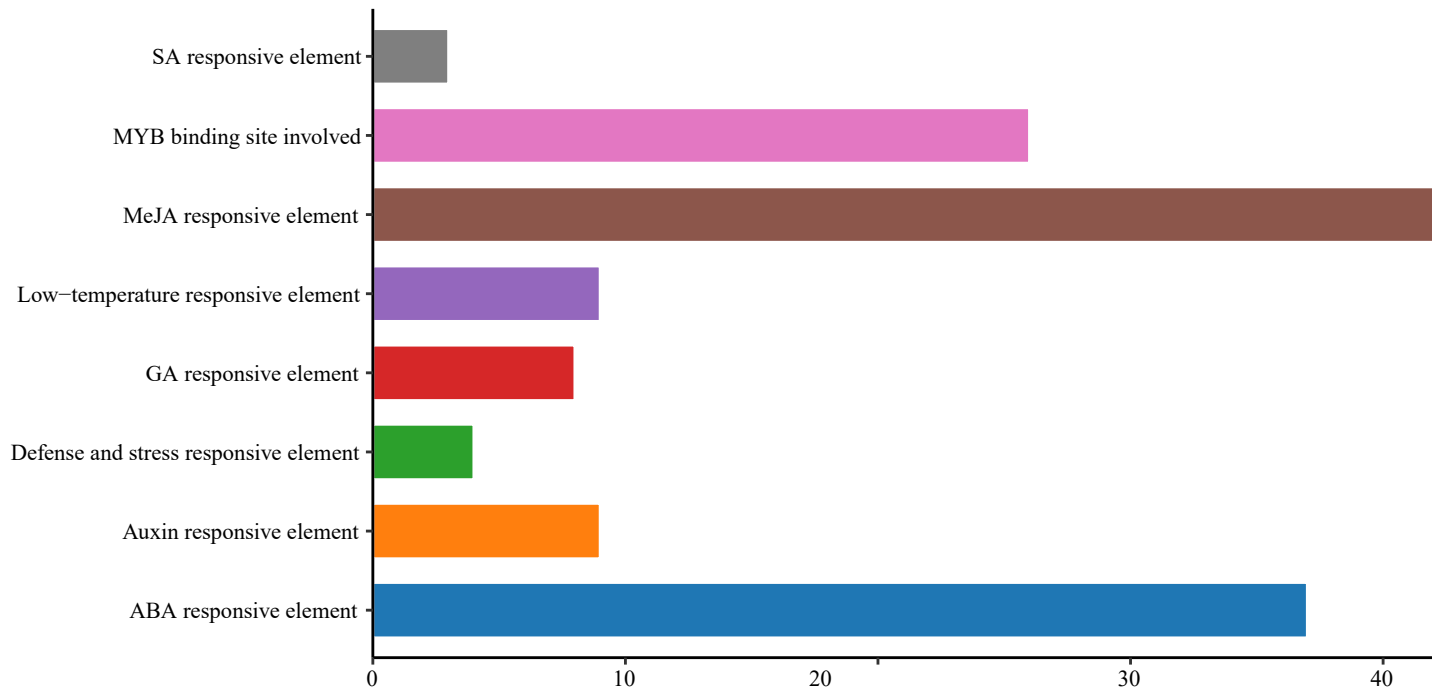

Figure S2: Number of *cis*-acting element

Supplement: Supplementary file 1 [file genes-14-00217-s001.zip › Figure S2.pdf]
